# Denosumab Combined With PARP Inhibitors and Chemoradiotherapy for Treating Triple Negative Breast Cancer With Bone Metastasis: A Case Report

**DOI:** 10.1002/cnr2.70463

**Published:** 2026-03-06

**Authors:** Li Liu, Lan Yu, Yu Wang, Hongmei Zhang, Xiaotao Zhang

**Affiliations:** ^1^ Department of Radiotherapy Qingdao Central Hospital, University of Health and Rehabilitation Sciences (Qingdao Central Hospital) Qingdao Shandong China

**Keywords:** bone metastasis, capecitabine, chemoradiotherapy, denosumab, homologous recombination deficiency, pamiparib, PARP inhibitors, progression‐free survival, skeletal‐related events, triple‐negative breast cancer

## Abstract

**Background:**

Triple‐negative breast cancer (TNBC) with bone metastasis is associated with poor prognosis and limited treatment options. Denosumab has shown efficacy in preventing skeletal‐related events, while PARP inhibitors have demonstrated promising activity in patients with homologous recombination deficiency.

**Case:**

We report a patient with TNBC and bone metastasis who received a combination treatment including denosumab, PARP inhibitors, and chemoradiotherapy. The treatment resulted in effective disease control and improvement of clinical symptoms. Imaging evaluation showed stabilization of bone lesions, and the patient tolerated the treatment well without severe adverse events.

**Conclusion:**

This case suggests that the combination of denosumab with PARP inhibitors and chemoradiotherapy may provide a potential therapeutic strategy for TNBC patients with bone metastasis. Further studies are warranted to validate the efficacy and safety of this combined treatment approach.

## 1 Introduction

Breast cancer is the most common malignancy among women, with an increasing incidence each year. Triple‐negative breast cancer (TNBC) remains the molecular subtype with the poorest prognosis. In 2020, there were approximately 416 000 new cases of breast cancer in China, ranking first among women, with TNBC accounting for about 15% of these cases. The 5‐year overall survival (OS) rate for patients with advanced TNBC is only 12.2% [1]. Around 50% of patients with advanced breast cancer have bone metastases, and approximately 70% of breast cancer deaths are confirmed to have bone metastases [2]. The survival period for breast cancer patients with bone metastases is significantly shortened. The 3‐year survival rate for patients with only bone metastases is 50.5%, with a median survival of 36 months [3]. Additionally, skeletal‐related events (SREs) such as pathological fractures and spinal cord compression due to bone metastases severely impact the quality of life and indirectly shorten the expected lifespan [4, 5]. This article reports, for the first time, a case of a TNBC patient with bone metastases who achieved long‐term progression‐free survival under combined treatment with denosumab and a PARP inhibitor, with good tolerability.

## 2 Case Presentation

The 71‐year‐old female patient underwent a modified radical mastectomy of the right breast on May 16, 2019 in Qingdao Central Hospital, University of Health and Rehabilitation Sciences (Qingdao Central Hospital). Postoperative pathology revealed grade 3 invasive ductal carcinoma (IDC) of no special type, measuring 2 × 2 × 1 cm, accompanied by high‐grade ductal carcinoma in situ (DCIS). Pathology also indicated low stromal tumor‐infiltrating lymphocytes (TIL), vascular invasion, and metastasis to one out of 20 examined axillary lymph nodes. Immunohistochemistry results showed ER (−), PR (−), Her‐2 (0), with a Ki‐67 positive rate of approximately 20%. The postoperative staging was pT1cN1aM0, Stage II.

The patient subsequently received four cycles of adjuvant chemotherapy with the AC regimen (Doxorubicin (Adriamycin) + Cyclophosphamide (Cytoxan)).

**FIGURE 1:**
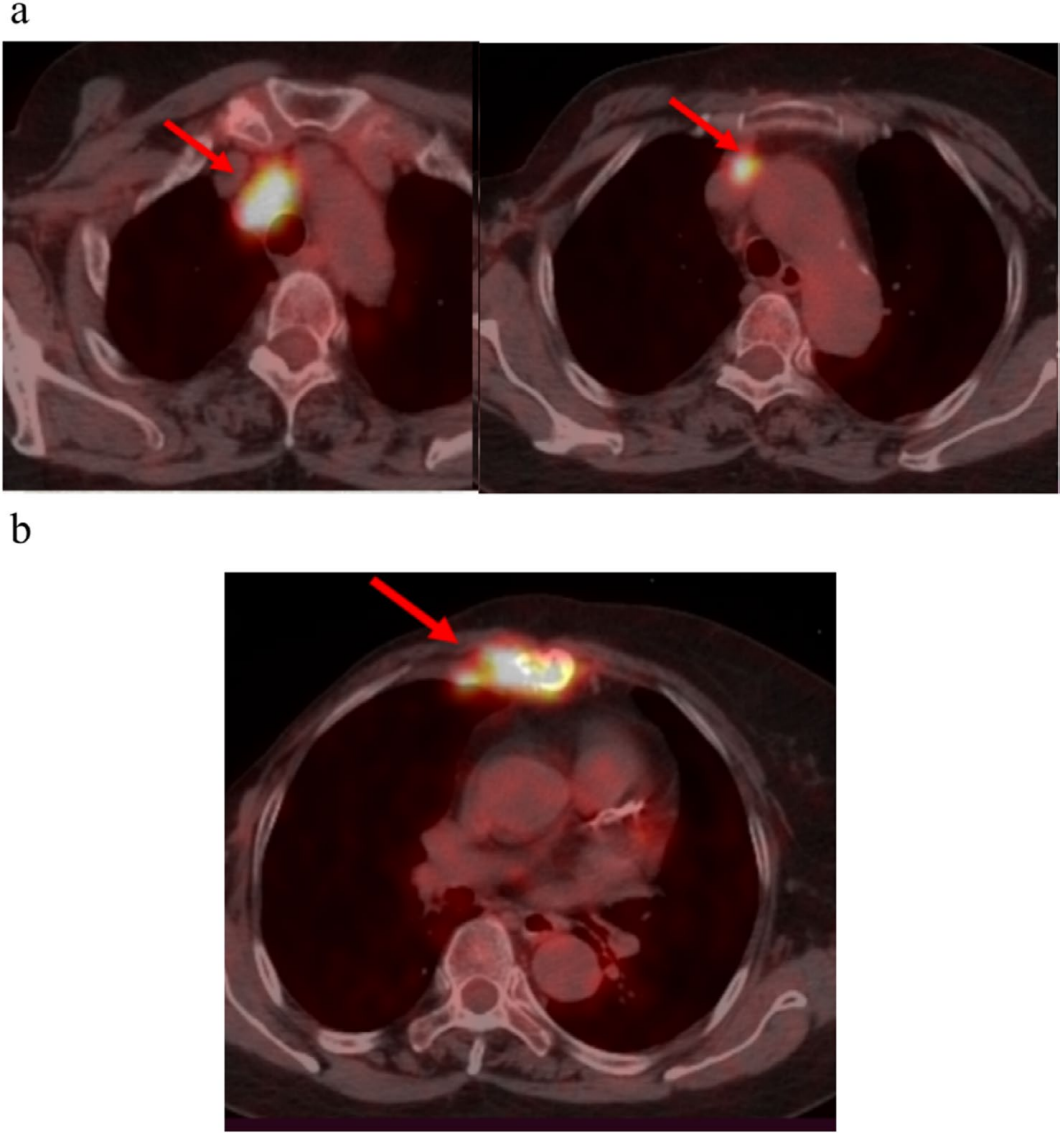
Metabolic activity in the right internal mammary region, mediastinum (prevascular and pretracheal spaces), right upper paratracheal lymph nodes, suggesting metastasis (a); osteolytic destruction in the sternum with increased metabolic activity, indicating sternal metastasis (b).

In January 2021, a follow‐up PET‐CT scan revealed metabolic activity suggesting metastasis in the right internal mammary region, mediastinum (prevascular and pretracheal spaces), and right upper paratracheal lymph nodes. Additionally, osteolytic destruction of the sternum with increased metabolic activity indicated possible sternal metastasis (Figure 1).

**FIGURE 2:**
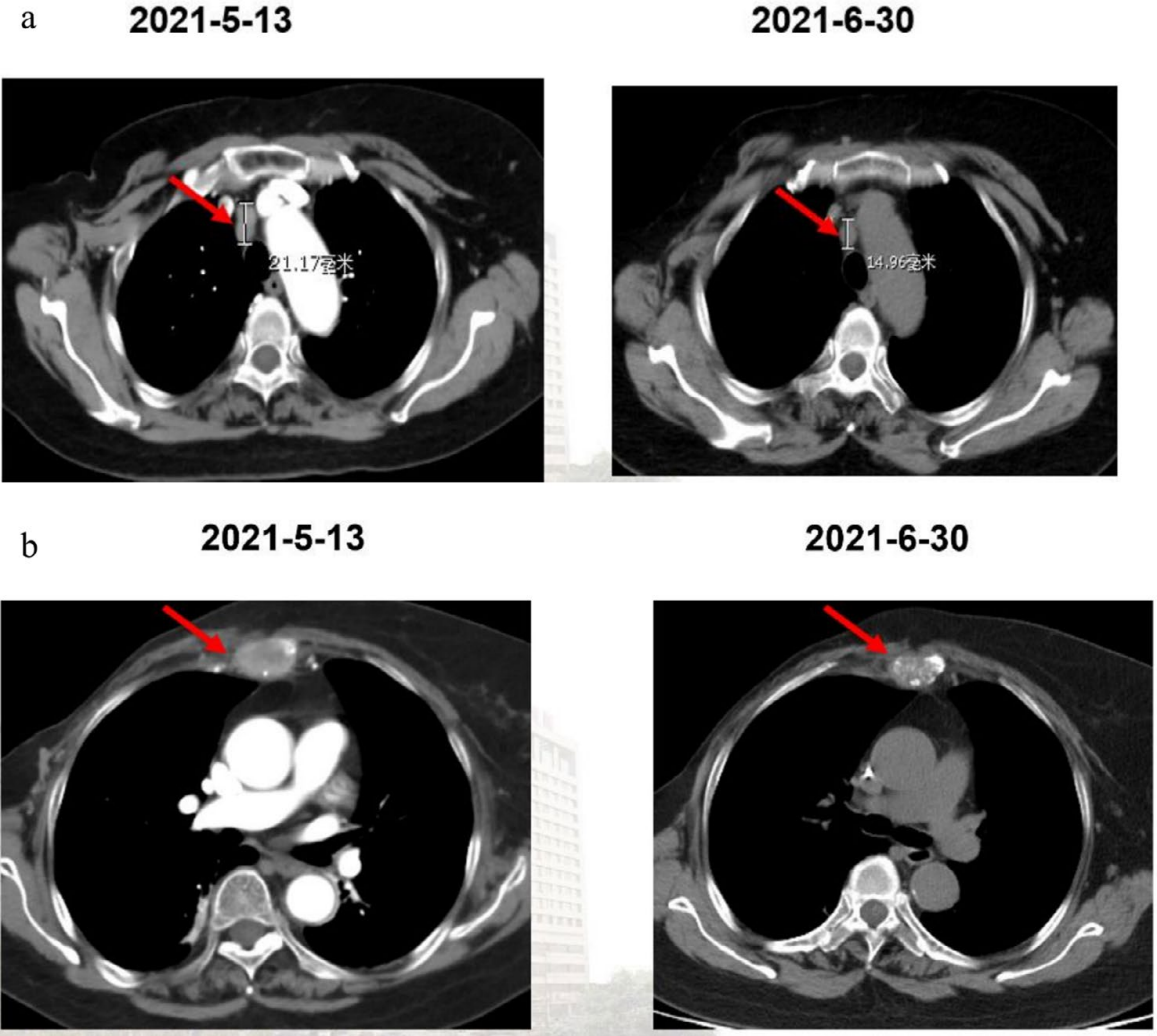
Response of mediastinal lymph nodes (reduced from 21.17 to 14.96 mm) after 2 cycles (a); sternum bone metastasis transformed from osteolytic to osteoblastic (b).

**FIGURE 3:**
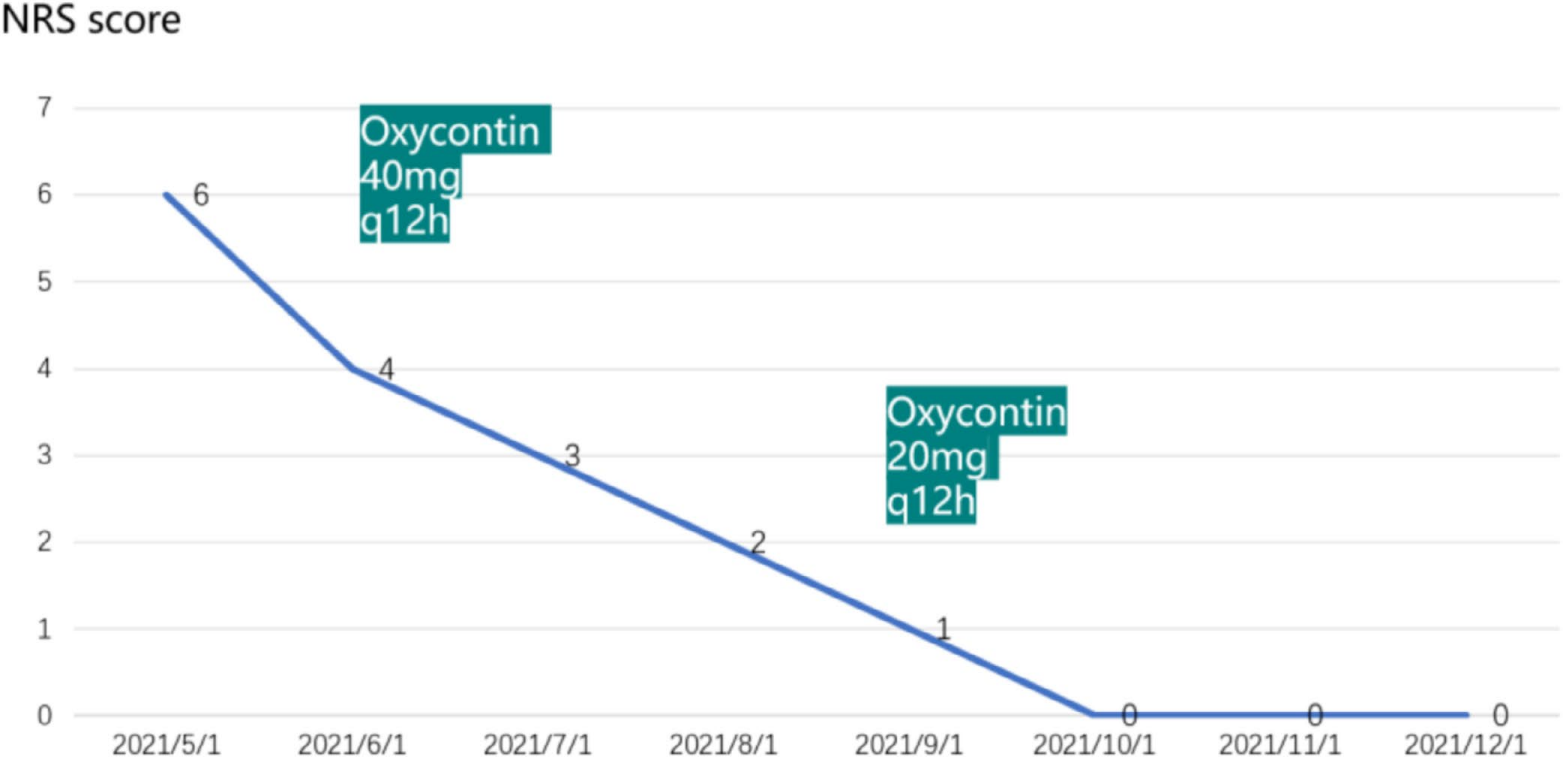
Change in NRS pain score.

In May 2021, due to worsening sternal pain, a chest CT scan confirmed mediastinal lymph node metastasis and osteolytic bone metastasis in the sternum. The patient then received four cycles of chemotherapy with nab‐paclitaxel (300 mg, every 3 weeks), starting May 15, 2021, and initiated monthly denosumab (120 mg) from May 17, 2021, continuing to date. After two cycles of treatment, a partial response (PR) was observed in the mediastinal lymph nodes, where they reduced from 21.17 to 14.96 mm in diameter, and the bone metastasis transformed from osteolytic to osteoblastic (Figure 2), with no skeletal‐related events (SREs) occurring, while the patient's NRS (Numerical Rating Scale) score improved from moderate–severe pain to no pain, allowing for a gradual reduction in pain medication (Figure 3).

**FIGURE 4:**
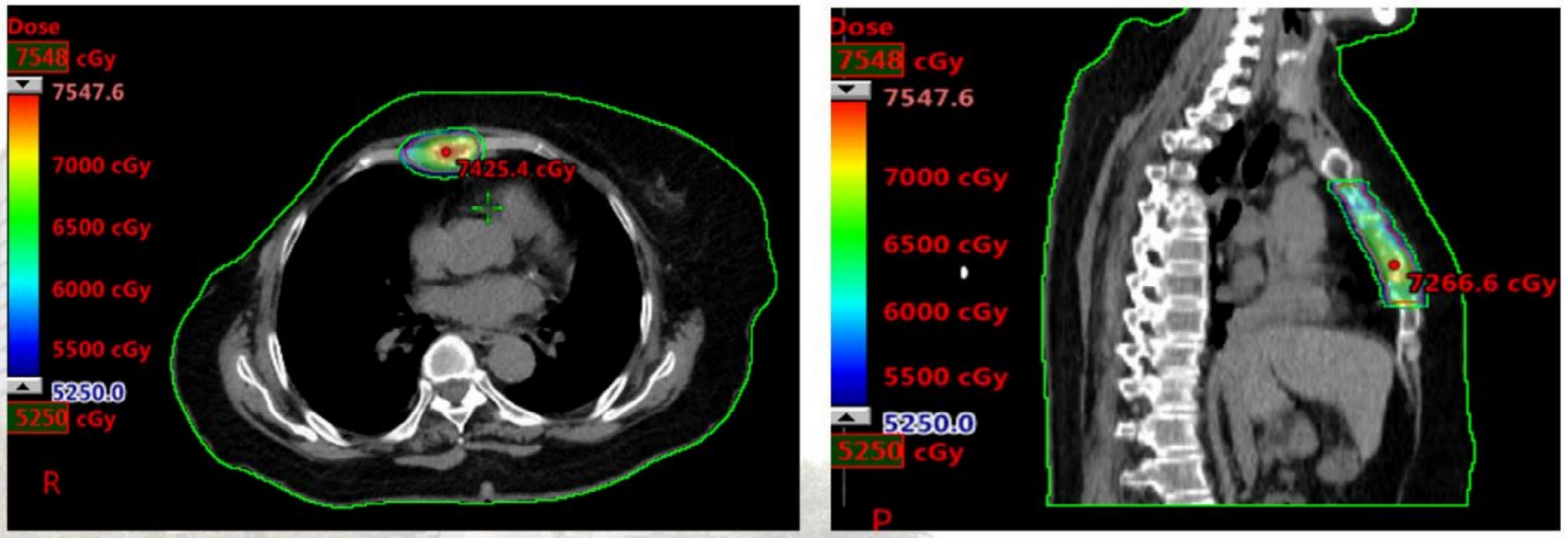
ARC technique was used to deliver hypofractionated radiotherapy to the sternal metastasis.

From July 12, 2021, the patient underwent palliative radiotherapy for the sternal metastasis using ARC technique, with a total dose of 52.5 Gy delivered in 15 fractions (3.5 Gy per fraction, covering 95% of the planned target volume) (Figure 4).

Deleterious germline alterations in BRCA1/2 genes are detected in approximately 5% of unselected patients with breast cancer [6, 7]. Several PARP inhibitors have been developed and investigated in patients with breast cancer. According to the NCCN Breast Cancer Guidelines, which recommend gBRCA1/2 testing for all patients with recurrent or metastatic breast cancer [8], genetic testing was performed on the patient's tissue, which revealed homologous recombination deficiency (HRD) positivity on August 4, 2021.

As the activity of PARP inhibitors relies on synthetic lethality in the context of an impaired HRR pathway, these agents have also been investigated in HRD or BRCAness tumors without gBRCA1/2 alterations. The combination of PARP inhibitors and chemotherapy has synergistic activity in clinical trials [9], thus the patient maintenance therapy with pamiparib (20 mg bid) and capecitabine (1.5 g bid) was initiated on September 13, 2021. This combination therapy continued until October 2022, after which the patient has been maintained on single‐agent pamiparib (see Table 1). Throughout the course of therapy, grade 2 neutropenia and moderate anemia occurred, which improved after symptomatic management. Routine biochemical tests during chemotherapy showed no significant abnormalities. Alanine aminotransferase (ALT) was 23 U/L (reference range: 7–40 U/L), aspartate aminotransferase (AST) 34 U/L (13–35 U/L), alkaline phosphatase (ALP) 70 U/L (50–135 U/L), and serum creatinine 46 μmol/L (41–81 μmol/L). Tumor markers, including carcinoembryonic antigen (CEA) and cancer antigen 125 (CA125), remained within normal reference ranges during treatment.

**TABLE 1:** Treatment regimens.

Drug	Usage and dosage	Starting and finishing date
Cyclophosphamide + Pegylated Liposomal Doxorubicin	Cyclophosphamide 1.0 g Day 1 + Pegylated Liposomal Doxorubicin 50 mg Day 1 (Cyclophosphamide: 600 mg/m^2^, every 3 weeks, for a total of 3 cycles; Pegylated Liposomal Doxorubicin: 35 mg/m^2^, every 3 weeks, for a total of 3 cycles)	June 1–July 12, 2019
Paclitaxel (albumin‐bound)	300 mg Day 1 (260 mg/m^2^, every 3 weeks, for a total of 6 cycles; dose reduction considered due to age and physical condition)	May 15–September 9, 2021
Denosumab	120 mg, subcutaneous injection, q4w	May 17, 2021–present
Radiotherapy	52.5 Gy/3.5 Gy/15 f	July 12–July 30, 2021
Pamiparib	20 mg bid	September 13, 2021–present
Capecitabine	1.5 g bid	September 13, 2021–October 1, 2022

As of now, the patient has achieved a progression‐free survival (PFS) of over 34 months.

## 3 Discussion

Studies revealed that up to 30% of patients with breast cancer develop SREs within 3 months if not promptly treated with bone‐modifying drugs after bone metastasis develops [10]. As such, bone‐modifying drugs are important for treating and preventing SREs in combination with anti‐tumor therapies [11]. An international, randomized, double‐blinded, double‐dummy, positive‐controlled study compared the effects of denosumab and zoledronate in delaying or preventing SREs in patients with breast cancer and bone metastasis [12]. Among the included 2046 patients with advanced breast cancer and bone metastasis, the occurrence time of the initial SRE in the denosumab‐treated group was significantly delayed compared to the group treated with zoledronate (not reached vs. 26.4 months, HR = 0.82, 95% CI: 0.71–0.95, *p* < 0.001). In addition, the denosumab group had an 18% reduced risk of initial skeletal complications, with lower incidences of acute phase response and renal adverse events. This study demonstrates the treatment efficacy of denosumab as well as its good safety and compliance in long‐term use. One of the main defining characteristics of pamiparib is that it is currently the only PARP inhibitor that is not a substrate of p‐glycoprotein (P‐gp). P‐gp is a transmembrane efflux pump that moves substrates from the inside to outside the cell. This means that once the drug enters the cell, it is not easily pumped out, allowing the intracellular drug concentration to remain relatively stable and thus ensuring its efficacy. This could be related to its superior Objective Response Rate (ORR) and Duration of Response (DOR). Additionally, this characteristic endows pamiparib with anti‐resistance properties, potentially reducing the likelihood of resistance. Therefore, metabolic interaction between osteoclasts and tumor cells contributes to resistance to DNA‐damaging agents, which can be blocked by combination treatment with PARP and osteoclast inhibitors to reduce bone metastatic burden [13]. Pamiparib might be a viable option for patients who are resistant to chemotherapy or other PARP inhibitors, although further clinical studies are needed to validate this.

## 4 Conclusion

This case highlights the effectiveness of combined therapy in controlling metastatic breast cancer, particularly bone metastasis. The use of pamiparib and capecitabine as maintenance therapy showed significant disease control, as evidenced by the prolonged PFS. The integration of denosumab in the treatment regimen contributed to the management of bone metastases, transforming osteolytic lesions to osteoblastic and preventing skeletal‐related events.

The patient's response to pamiparib, a PARP inhibitor, in the context of HRD positivity underscores the importance of personalized medicine in treating advanced breast cancer. Furthermore, the utilization of ARC technique for radiotherapy provided targeted treatment to the sternal metastasis with minimal adverse effects.

Overall, this case study demonstrates the potential benefits of a multidisciplinary approach in managing complex cases of breast cancer with bone metastasis. It also emphasizes the roles of targeted therapy, chemotherapy, radiotherapy, and bone‐modifying agents in providing effective and suitable treatment options for these patients.

## Author Contributions

Li Liu contributed to the conception and design of the study, data collection, data analysis and interpretation, and drafting of the manuscript. Lan Yu contributed to clinical data acquisition and interpretation. Yu Wang contributed to radiological evaluation and data interpretation. Hongmei Zhang contributed to critical revision of the manuscript for important intellectual content. Xiaotao Zhang contributed to study supervision and final approval of the manuscript. All authors read and approved the final manuscript.

## Funding

The authors have nothing to report.

## Consent

We confirm that informed written consent was obtained from the patient for the publication of this case report and any accompanying images or data. The patient has voluntarily agreed to the publication and fully understands that the report may be available online and accessible to a broad audience.

## Conflicts of Interest

The authors declare no conflicts of interest.

## Data Availability

The data that support the findings of this study are available from the corresponding author upon reasonable request.
